# Epidemiology of Hypertension in Serbia: Results of a National Survey

**DOI:** 10.2188/jea.JE20110077

**Published:** 2012-05-05

**Authors:** Grujić Vera, Dragnić Nataša, Kvrgić Svetlana, Šušnjević Sonja, Grujić Jasmina, Travar Sonja

**Affiliations:** 1 Institute of Public Health of Vojvodina, Novi Sad, Serbia; 2 Institute for Transfusion, Novi Sad, Serbia

**Keywords:** hypertension, adult population, Serbia

## Abstract

**Background:**

We evaluated the prevalence of high blood pressure and the level of awareness, treatment, and control of hypertension in a Serbian population.

**Methods:**

A cross-sectional study of an adult population was carried out across Serbia in 2006. The study involved 14 204 adults aged 20 years or older. Interviews and measurements of blood pressure were performed at participants’ homes.

**Results:**

Overall, 47% of the Serbian adult population had hypertension: 25.3% had stage 1 hypertension and 18.1% had stage 2 hypertension. Only 58.0% of the hypertensive population were aware that they had the disease, and 60.4% were receiving medical treatment. Among those receiving medical treatment, only 20.9% had a blood pressure within the normal range. One in 10 participants with hypertension were not treated because, among other reasons, they thought treatment was unnecessary (55.3%) or they lacked money for medication (19.3%).

**Conclusions:**

The prevalence of undiagnosed and untreated hypertension is high in the adult population of Serbia. Further action is required to hasten detection and treatment of high blood pressure. Attention should be directed toward educational programs that improve knowledge, attitudes, and awareness of hypertension among adults.

## INTRODUCTION

Cardiovascular disease (CVD) is the most common cause of death in developed countries, and hypertension is one of the most treatable and modifiable risk factors for CVD.^1^^,^^2^ Hypertension (or high blood pressure, HBP) is a major problem worldwide, and approximately 1 billion people have the condition.^3^ The 2002 World Health Report identified HBP as the third most important cause of disability and a potent risk factor for heart disease (as well as for stroke and renal disease), the leading cause of death worldwide.^4^^–^^8^

Many people have HBP for a considerable period of time before it is diagnosed.^7^ Pharmacologic treatment of HBP decreases the risk of CVD, including stroke, coronary heart disease, and renal insufficiency.^9^^,^^10^ Nonpharmacologic interventions are also beneficial for preventing and treating HBP.^11^^–^^13^

Patients who are aware that HBP reduces life expectancy have better adherence to medication and better blood pressure control than do populations without such awareness.^14^^,^^15^ Our data from 2000 indicate that half of the studied population of Serbia with HBP were aware of the disease, while 34.5% were taking prescribed medication. Similarly, blood pressure was well controlled in only 2.6% of the studied population. Moreover, the prevalence of HBP in Serbia was 44.5% in 2000, and a further increase was expected.^16^ Therefore, it is necessary to take action and decrease such unfavorable trends. To address this, the present study assessed the prevalence of HBP, and the awareness, treatment, and control of HBP, in Serbia.

## METHODS

The Ministry of Health of the Republic of Serbia carried out a National Survey of the population of Serbia in 2006. The study was a follow-up of a baseline study conducted in 2000.^16^ The studied population consisted of men and women aged 20 years or older. People located in retirement homes, social institutions, prisons, and psychiatric institutions were excluded from the study. The sample frame encompassed all households within the 2002 Census of Serbia. A stratified 2-stage sample of the population was used. In Serbia, 3 geographic areas were identified: the Province of Vojvodina, Belgrade, and Central Serbia. A further stratum classification was urban vs rural area of residence. In the first stage of 2-stage sampling, census circles were selected (total, 675 census circles) by probability proportional sampling. In the second stage, households were selected by a linear method of sampling, with a random beginning and an equal selection interval, by random sampling without replacement. Lists of households in all chosen census circles were updated before the selection of households. After updating every census circle, 10 households and 3 reserves were selected from every list.

A sample was selected to represent the population across Serbia. It also allowed reliable statistical evaluation of all characteristics with a frequency of appearance greater than 5%. For the total population, a relative error of 5% of evaluated proportions was anticipated. Concerning the requirements for precise evaluation and the level of evaluation reliability obtained, we selected the number of examinees that enabled sufficient sample size for each region. The poll included 6156 households in Serbia (14 204 of the adult population aged 20 or older). HBP was found in 6642 of the adult population. Blood pressure measurement was performed with the participant in a sitting position after they had rested for at least 5 minutes. Three readings were taken, with a 1-minute interval between measurements. The mean of the first 2 readings was recorded. However, if the difference between the first and second reading was greater than 10 mm Hg, then the mean of the 2 closest measurements was used.^17^

HBP was defined as an average systolic blood pressure (SBP) of 140 mm Hg or higher, a diastolic blood pressure (DBP) of 90 mm Hg or higher, or use of antihypertensive medication.^17^^,^^18^ Severity of HBP was classified into 2 stages. Stage 1 was defined as an SBP of 140 to 159 mm Hg or a DBP of 90 to 99 mm Hg; stage 2 was defined as an SBP of 160 mm Hg or higher or a DBP of 100 mm Hg or higher.^19^

Categories of HBP were defined as untreated HBP (no use of medication and no dietary intervention) and treated HBP (use only of a prescribed medication for management of HBP at the time of the interview). Control of HBP was defined as pharmacologic treatment of HBP resulting in an SBP lower than 140 mm Hg and a DBP lower than 90 mm Hg. Assessment of reasons for not being treated for HBP was done by questionnaire and a multiple choice question with 4 responses: no need, no medication, no money, or other (specify). Awareness of HBP was defined as a participant with HBP who responded affirmatively to the question “Have you ever been told by a doctor or other health professional that you have high blood pressure?”.^20^^,^^21^ The sociodemographic variables included age group (20–34, 35–44, 45–54, 55–64, and ≥65 years), sex, and type of settlement (urban and rural).

### Statistical analysis

The obtained data were statistically analyzed using SPSS 17 for Windows. Results are reported as mean ± standard deviation (SD), confidence interval (CI), and proportion. Differences in frequency and sample means were tested by the chi-square test, ANOVA, and Student *t*-test (a *P* value <0.05 was considered to be statistically significant). All reported *P* values are 2-tailed.

## RESULTS

### Hypertension prevalence

The mean age was 51.0 years for women and 49.5 years for men. Mean age was higher (*P* < 0.001) in rural than in urban areas, regardless of sex (men: 50.6 vs 48.3; women: 52.1 vs 50.0). Overall, the prevalence of HBP was 46.8% (95% CI 45.9–47.6)—47.3% in men (46.1–48.6) and 46.2% in women (45.1–47.7)—and was higher in each successive age group in both sexes. HBP prevalence varied by type of settlement (ie, urban vs rural): it was significantly higher (*P* < 0.001) in rural than in urban areas (49.4% vs 44.3%; Table 1).

**Table 1.** Prevalence of hypertension in the Serbian adult population by sex, age, and type of settlement

Type of settlement		Men						Women						All adults
		
		20–34	35–44	45–54	55–64	65+	Total	20–34	35–44	45–54	55–64	65+	Total	
Urban*	*n*	834	593	684	564	671	3346	904	683	759	717	941	4004	7350
	%	19.4	32.4	50.4	61.2	71.1	45.5	6.3	19.5	43.6	67.2	77.7	43.3	44.3
Rural*	*n*	735	538	659	519	860	3311	739	533	644	552	1075	3543	6854
	%	21.1	36.1	48.4	66.3	72.0	49.3	6.9	23.8	49.4	73.2	79.6	49.6	49.4

Total*	*n*	1569	1131	1343	1083	1531	6657	1643	1216	1403	1269	2016	7547	14 204
	%	20.2	34.1	49.4	63.6	71.6	47.3	6.6	21.4	46.3	69.8	78.7	46.2	46.8

*All differences among age groups in the prevalence of hypertension were statistically significant (*P* < 0.001) in men and women.

### Mean blood pressure level

The mean blood pressure of all adults was 135.2/82.4 mm Hg (SBP: 95% CI, 134.9–135.6; DBP: 95% CI, 82.2–82.6). Mean SBP was higher among men (136.3 ± 19.0) than among women (134.3 ± 23.5) and increased with age in both sexes. It was higher in rural than in urban areas, regardless of sex (men: 137.3 ± 20 vs 135.3 ± 18.0 mm Hg; women: 136.3 ± 24.4 mm Hg vs 132.6 ± 22.5 mm Hg; Table 2). Mean DBP was higher in rural areas than in urban areas among women (82.6 ± 13.6 vs 80.6 ± 12.7 mm Hg) and men (83.8 ± 12.4 vs 83.1 ± 11.1 mm Hg).

**Table 2.** Mean systolic and diastolic blood pressure in the Serbian adult population by sex, age, and type of settlement

			Men						Women						All adults
				
			20–34	35–44	45–54	55–64	65+	Total	20–34	35–44	45–54	55–64	65+	Total	
SBP	Urban*	Mean	126.2	129.8	136.6	140.5	145.7	135.3	115.7	121.4	132.4	142.4	149.5	132.6	133.8
		SD	11.0	14.0	17.4	18.4	20.8	18.0	12.3	15.7	19.9	20.8	21.5	22.5	20.6
	Rural*	Mean	127.3	121.4	136.0	144.0	147.0	137.3	116.7	123.5	135.4	146.5	151.4	136.3	136.8
		SD	12.5	15.7	18.7	21.5	22.1	20.0	11.6	15.9	20.3	22.8	24.5	24.4	22.4
	Total*	Mean	126.7	130.3	136.6	142.2	146.4	136.3	116.2	122.3	133.8	144.2	150.5	134.3	135.2
		SD	11.7	14.4	18.1	20.0	21.5	19.0	12.0	15.8	20.1	21.8	23.1	23.5	21.5

DBP	Urban*	Mean	79.1	82.2	85.5	85.5	84.5	83.1	72.3	76.5	82.1	86.2	85.9	80.6	81.7
		SD	8.9	10.4	11.8	10.8	12.2	11.1	8.9	10.9	12.7	11.9	12.6	12.7	12.1
	Rural*	Mean	79.0	82.5	85.0	87.3	85.7	83.8	73.1	78.3	83.8	88.2	87.6	82.6	83.2
		SD	9.0	10.9	12.1	15.6	12.4	12.4	9.2	11.5	13.1	13.0	13.4	13.6	13.0
	Total*	Mean	79.0	82.3	85.2	86.4	85.2	83.5	72.6	77.3	82.9	87.1	86.8	81.5	82.4
		SD	9.0	10.6	12.0	13.4	12.3	11.8	9.1	11.2	12.9	12.4	13.0	13.2	12.6

Abbreviations: SBP, systolic blood pressure, DBP, diastolic blood pressure.

*All differences among age groups were statistically significant (*P* < 0.001) in both sexes.

### Distribution of blood pressure

Sex- and age-specific distributions of blood pressure in rural and urban areas are shown in Table 3. Normal blood pressure was identified in 53.2% of the total study population (52.7% of men and 53.8% of women). HBP was well controlled in 3.4% of adults, and more often in women than men (4.3% vs 2.4%) regardless of type of settlement. Stage 1 HBP was identified in 25.3% of participants and stage 2 HBP was noted in 18.1% of participants. These proportions increased with age regardless of sex or type of settlement.

**Table 3.** Percentage distribution of blood pressure level in the Serbian adult population by sex, age, and type of settlement

*Type of settlement* Blood pressure level	Men						Women						All adults
	
	20–34	35–44	45–54	55–64	65+	Total	20–34	35–44	45–54	55–64	65+	Total	
Urban*													
Normotensive	80.6	67.6	49.6	38.8	28.9	54.5	93.7	80.5	56.4	32.8	22.3	56.7	55.7
Controlled	0.6	1.5	1.6	5.0	4.0	2.4	0.2	2.5	4.2	8.2	6.6	4.3	3.4
Hypertension I	15.1	24.1	31.3	36.0	38.9	28.3	5.2	11.9	22.4	32.1	35.8	21.6	24.7
Hypertension II	3.8	5.8	17.6	20.2	28.2	14.8	0.9	5.2	17.0	26.9	35.3	17.4	16.2

Rural*													
Normotensive	78.9	63.9	51.6	33.7	28.0	50.7	93.1	76.2	50.6	26.8	20.4	50.4	50.6
Controlled	0.8	0.7	2.7	3.5	4.2	2.5	0.9	2.1	3.9	6.3	6.9	4.3	3.4
Hypertension I	17.0	24.7	29.3	34.7	34.2	27.9	5.0	15.0	29.5	33.7	34.0	24.2	26.0
Hypertension II	3.2	10.6	16.4	28.2	33.6	33.6	0.9	6.8	16.0	33.2	38.8	21.1	20.0

Total*													
Normotensive	79.8	65.9	50.6	36.4	28.4	52.7	93.4	78.6	53.7	30.2	21.3	53.8	53.2
Controlled	0.7	1.1	2.2	4.2	4.1	2.4	0.5	2.3	4.1	7.4	6.7	4.3	3.4
Hypertension I	16.0	24.4	30.3	35.4	36.3	28.1	5.1	13.2	25.7	32.8	34.8	22.8	25.3
Hypertension II	3.5	8.6	16.9	24.0	31.3	16.8	1.0	5.8	16.6	29.6	37.1	19.1	18.1

*All differences were statistically significant (*P* < 0.001) in men and woman.

### Awareness, treatment, and control of hypertension

Participant awareness of HBP is shown in Table 4. Only 58.0% of all participants with hypertension were aware of the condition. Women had greater awareness of HBP than did men (66.5% vs 48.6%), regardless of age group or type of settlement. Among all hypertensive individuals who were aware of their condition, 60.4% were receiving treatment. The percentage of hypertensive participants receiving treatment was higher among women (63.2%) than among men (56.2%, *P* < 0.001) and in rural areas (61.6%) than in urban areas (59.2%, *P* < 0.001). The proportion of participants receiving treatment for HBP increased with age regardless of sex or type of settlement. The proportion of participants with hypertension who were not treated was higher among men than among women (14.2% vs 5.6%, *P* < 0.001). The percentage of participants with untreated hypertension significantly decreased with age in men and women in urban and rural areas. The most frequent reasons reported for lack of treatment were no need for treatment (55.3%) and not enough money to pay for medication (19.3%). Reasons for nontreatment significantly differed by age (*P* = 0.008) and sex (*P* < 0.001). The percentage of people who thought they had no need for treatment decreased with advancing age (69.2% among those aged 20–34 years vs 46.7% among those aged 65 years or older), while the percentage of people who reported not having enough money for medication increased with age (3.8% among those aged 20–34 years vs 33.7% among those aged 65 years or older). The percentage of adults who were aware of HBP but had not received treatment because they thought it was unnecessary was higher in men (59.2%) than in women (49.2%). Women were more likely than men to report that they had no money for treatment (29.8% vs 13.1%). There were differences in the reasons for nontreatment with regard to urban and rural residence (no need: 57.9% vs 53.4%; no money: 14.5% vs 23.6%, data not shown in table).

**Table 4.** Proportions of participants aware of hypertension, treated for hypertension, and with adequately controlled hypertension, by sex, age, and type of settlement

		Men						Women						All adults
			
		20–34	35–44	45–54	55–64	≥65	Total	20–34	35–44	45–54	55–64	≥65	Total	
Aware*	Urban	17.9	34.9	50.4	57.7	62.1	50.3	23.2	45.9	62.5	76.0	75.7	69.3	60.4
	Rural	15.5	26.0	43.6	55.8	58.4	47.1	35.3	40.9	58.5	66.6	69.6	63.8	55.8
	Total	16.7	60.5	47.1	56.7	60.0	48.6	29.0	43.5	60.6	71.7	72.4	66.5	58.0

Treated^a,^*	Urban	34.5	52.2	45.4	56.3	60.1	54.1	53.8	60.7	59.2	60.7	65.2	62.5	59.2
	Rural	58.3	48.0	53.2	57.8	61.8	58.2	55.6	55.8	58.1	64.6	66.4	63.9	61.6
	Total	45.3	50.4	48.9	57.0	61.0	56.2	54.8	58.4	58.7	62.3	65.8	63.2	60.4

Untreated^b,^*	Urban	41.4	28.4	21.8	8.5	6.4	13.7	23.1	14.8	9.2	3.3	2.4	4.7	8.2
	Rural	29.2	30.0	20.1	12.5	10.8	14.8	27.8	15.4	12.4	4.9	4.0	6.5	9.9
	Total	35.8	29.1	21.1	10.5	8.8	14.2	25.8	15.0	10.7	4.0	3.2	5.6	9.0
Controlled^c,^*	Urban	50.0	25.7	13.9	25.0	15.2	19.3	28.6	45.9	26.2	26.2	17.2	22.9	21.7
	Rural	42.9	16.7	24.3	16.2	16.1	18.4	70.0	37.9	23.1	20.2	18.7	21.3	20.1
	Total	45.8	22.0	19.0	20.6	15.7	18.8	52.9	42.4	24.8	23.6	18.0	22.1	20.9

^a^Number of people with hypertension who were aware of their condition and receiving medical treatment.

^b^Number of people with hypertension who were aware of their condition and untreated (either by medication, diet, or a combination thereof).

^c^Number of medically treated people with hypertension who were normotensive.

*All differences among age groups were statistically significant (*P* < 0.001) in urban-dwelling men and women. Differences were also significant among rural dwellers (except for controlled^c^ in men) and in the total population.

Among participants with hypertension receiving treatment, only 20.9% had well-controlled blood pressure (18.8% of men and 22.1% of women). The proportion of well-controlled HBP decreased with age regardless of sex or type of settlement.

## DISCUSSION

It is recognized that the high prevalence of HBP has contributed to the present global pandemic of CVD, which is responsible for 30% of all deaths worldwide.^21^ The prevalence of HBP varies widely among different populations and is somewhat dependent on factors such as race, lifestyle, and degree of urbanization. These differences may reflect the effects of dynamic interactions among genetic, demographic, sociocultural, and economic factors.^22^ The prevalence in adult populations worldwide varies from 5.2% to 70.7%.^23^^,^^24^ Existing data on the prevalence of HBP indicate that the average prevalence of HBP in Europe is 44.2%, as compared with 27.6% in North America. HBP prevalence was highest in Germany (55%), Finland (49%), Spain (47%), England (42%), Sweden (38%), and Italy (38%).^1^^,^^9^^,^^25^^–^^27^ In Serbia, the prevalence of HBP increased from 44.5% in 2000 to 46.8% in 2006.

Some studies suggest that HBP is more prevalent in men than in women.^28^^,^^29^ In other studies, the prevalence of HBP is higher in women than in men,^30^^,^^31^ whereas some studies no sex difference in prevalence.^22^ We found no difference in HBP prevalence between men and women, and the same results were obtained in a Serbian survey conducted in 2000.^16^ Age is strongly associated with HBP. Almost 1 in 4 adults aged between 35 and 44 years and every second person aged between 45 and 54 years in Serbia has HBP. More than 75.0% of adults aged 65 or older were hypertensive. An increase in HBP prevalence with age has been found in all regions of the world.^32^^,^^33^

Analysis of HBP in 2000 and 2006 showed that the prevalence of HBP was higher among adults in rural as compared with urban areas of Serbia, regardless of sex.^16^ Differences in HBP prevalence in rural and urban areas can be partly explained by older age (51.3 vs 49.2 years), lower educational level (61.3% of rural dwellers had a primary education level or lower, ie, elementary, incomplete, or no schooling), lower socioeconomic status (68.7% were unemployed), and statistically higher prevalences of unhealthy lifestyle behaviors in rural as compared with urban areas.^34^ The percentage of overweight people (body mass index >25) was also higher in rural than in urban areas (65.2% vs 61.3%), as was use of animal fats in cooking (64.6% vs 20.7%).^34^^,^^35^

Mean SBP and DBP were higher in European countries than in Canada and the United States, with an average of 136/83 mm Hg in Europe and 127/77 mm Hg in North America.^1^ The National Health and Nutrition Examination Survey (NHANES III) reported higher mean SBP and DBP and higher overall arterial pressure in normotensive and hypertensive men than in women.^36^ In Serbia, mean blood pressure was close to the European average and was higher in men than in women. As compared with participants aged 20 to 34 years, SBP increased by 9.9 mm Hg in men aged 45 to 54 years and by 17.6 mm Hg in women of the same age. As compared with participants aged 45 to 54, mean SBP increased in men by a further 9.8 mm Hg and in women by a further 16.7 mm Hg among those aged 65 or older. Mean SBP and DBP values were higher among men and women living in rural areas.

In European countries, average HBP control was lower (26.8%) than in North America (44.4%), the United States (52.5%), and Canada (36.3%).^1^ In Europe, awareness of hypertension varied from 46% in Portugal to 60% in Greece.^37^ Similar findings were reported in other research.^9^^,^^38^ NHANES III showed that the percentage of persons who are aware of their HBP was a rough proxy for the percentage of those who were being treated or treated and controlled.^9^ Although there has been significant progress in increasing awareness, detection, treatment, and control of HBP, clinical experience and research indicate that 50% to 75% of people who are diagnosed and treated for HBP do not have adequate blood pressure control.^39^^–^^42^ In 2006, 3.4% of all examinees used their prescribed medication and had well-controlled blood pressure, as compared with 2.6% in 2000.^16^

In 2006, hypertension was well controlled in only one-fifth of treated participants in Serbia. In addition, HBP was more frequently well controlled in women than in men and among those living in urban than in rural areas. The proportion of participants with well-controlled HBP decreased with age regardless of sex or type of settlement, which results in higher mean blood pressure. A possible explanation for this could be the increased number of older adults with severe HBP (stage 2). Severe HBP was found in 3.5% and 1.0% of young men and women (age 20–34 years) and in 31.3% and 37.1% of older men and women (age ≥65 years). There were urban–rural differences in reasons for nontreatment: in rural regions, a higher proportion of participants claimed that lack of money was the reason for nontreatment. The data also showed that there were significantly more households with low financial status in rural than in urban regions (65.1% vs 23.3%). This finding confirms previous evidence that financial cost is an important reason for untreated hypertension.^43^

HBP is important not only because of its high prevalence, but because it is a major modifiable risk factor for CVD and kidney disease. It is only one of several proven major modifiable risk factors for CVD.^44^ Therefore, a comprehensive approach is needed that focuses on several interrelated risks to health, including HBP, tobacco use, overweight and obesity, physical inactivity, poor diet, and diabetes mellitus.^45^

Although, our study has provided much-needed information on HBP and has filled a 7-year void in the knowledge of many risk factors associated with HBP (smoking, physical inactivity, use of alcohol, obesity), analysis of risk factors and their relationship with HBP was outside the scope of the present research. Such analysis should be undertaken in prospective studies in Serbia.
